# Fertility Protection in Female Cancer Patients: From Molecular Mechanisms of Gonadotoxic Therapies to Pharmacotherapeutic Possibilities

**DOI:** 10.3390/ijms26157314

**Published:** 2025-07-29

**Authors:** Weronika Zajączkowska, Maria Buda, Witold Kędzia, Karina Kapczuk

**Affiliations:** 1 Division of Gynaecology, Poznan University of Medical Sciences, Polna 33, 60535 Poznan, Poland; 73465@student.ump.edu.pl (W.Z.); witold.kedzia@poczta.fm (W.K.); 2 Doctoral School, Poznan University of Medical Sciences, Bukowska 70, 60812 Poznan, Poland; 3 Gynaecological and Obstetric Clinical Hospital of Poznan University of Medical Sciences, Polna 33, 60535 Poznan, Poland; maria.buda@gpsk.ump.edu.pl

**Keywords:** chemotherapy, radiotherapy, gonadotoxicity, fertoprotective agents, fertility preservation, oncofertility

## Abstract

Chemotherapeutic agents and radiotherapy are highly effective in treating malignancies. However, they carry a significant risk of harming the gonads and may lead to endocrine dysfunction and reproductive issues. This review outlines the molecular mechanisms of gonadotoxic therapies, focusing on radiation, alkylating agents, and platinum compounds. It discusses the loss of PMFs due to gonadotoxic exposure, including DNA double-strand breaks, oxidative stress, and dysregulated signaling pathways like PI3K/PTEN/Akt/mTOR and TAp63-mediated apoptosis. Furthermore, it explores strategies to mitigate gonadal damage, including GnRH agonists, AMH, imatinib, melatonin, sphingolipid metabolites, G-CSF, mTOR inhibitors, AS101, and LH. These therapies, paired with existing fertility preservation methods, could safeguard reproductive and hormonal functions and improve the quality of life for young cancer patients. Despite the progress made in recent years in understanding gonadotoxic mechanisms, gaps remain due to questionable reliance on mouse models and the lack of models replicating human ovarian dynamics. Long-term studies are vital for wider analyses and exploration of protective strategies based on various animal models and clinical trials. It is essential to verify that these substances do not hinder the anti-cancer effectiveness of treatments or cause lasting DNA changes in granulosa cells, raising the risk of miscarriages and infertility.

## 1. Introduction

Over the past 20 years, advancements in cancer treatment have increased the five-year survival rate for pediatric cancer patients to nearly 80% [1]. However, up to 75% of these survivors may experience late side effects from their treatments, which can significantly impact their well-being [2]. Consequently, improvements in the long-term quality of life after cancer treatment, particularly regarding fertility preservation, have become the subject of scientific research.

Chemotherapy and radiotherapy in female cancer patients can lead to ovarian atrophy and compromise the blood supply to the ovaries [3]. Radiation can reduce uterine volume and elasticity, damage musculature and the endometrium, and decrease vasculature [4]. A history of radiation therapy has been associated with adverse pregnancy outcomes, including accreta-spectrum disorders, preterm birth, and intrauterine growth restriction [5]. Additionally, irradiation to any part of the hypothalamic–pituitary–ovarian (HPO) axis can cause hypogonadism [6]. Chemotherapy alone may also negatively affect central hormonal regulation and cause hypothalamic–pituitary dysfunction, resulting in growth hormone deficiency, central hypothyroidism, precocious puberty, and/or gonadotropin deficiency, leading to various endocrine dysfunctions and reproductive challenges [7].

The extent of gonadotoxicity depends on several factors, including the type of therapy administered, the patient’s age, the treatment regimen, the cumulative dose of chemotherapy, the dosage, and the location of the radiation field. Women aged 35 and older exhibit nearly double the incidence of post-chemotherapy amenorrhea compared to younger women. This disparity is primarily due to differences in ovarian reserve; therefore, the impact of follicle loss is significantly more pronounced in older patients with a lower initial ovarian reserve [8]. Alkylating agents such as cyclophosphamide (Cy) and busulfan, along with platinum-based drugs like cisplatin (Cs) and carboplatin, are the most toxic chemotherapy agents for the ovaries. Pelvic, abdominal, and total body irradiation are the most harmful types of radiation to the gonads [9].

Damage caused by chemotherapy and radiotherapy can vary, ranging from partial damage to premature ovarian insufficiency (POI) and infertility. Almost all chemotherapeutic agents induce DNA alterations in granulosa cells and/or oocytes, leading to the apoptosis of growing follicles or the survival of mutagenic oocytes, resulting in temporary amenorrhea during and after chemotherapy treatment [10]. Menstruation usually occurs within one year following the completion of treatment, but it can also happen more than two years after the end of therapy [11]. POI may be diagnosed months or years after oncological treatment and is characterized by amenorrhea lasting longer than four months and elevated follicle-stimulating hormone (FSH) levels in women before the age of 40 [12]. However, it can also manifest early in childhood with the absence of spontaneous puberty or menstruation, or it may appear later, resulting in a shortened period of normal reproductive and hormonal function in the ovaries. This can lead to various adverse health outcomes, including an increased risk of cardiovascular disease, osteoporosis, depression, or anxiety [13].

In 2025, over 100 million women worldwide may be at risk of gonadotoxic effects following oncology therapy and could seek fertility preservation [14]. Cancer diagnosis and treatment can significantly affect the chances of becoming pregnant. This likelihood can range from very low to nearly 100%. Consequently, cancer survivors may have lower birth rates post-treatment [9]. Patients affected by infertility may experience a substantial decline in their quality of life, which often serves as a barrier to achieving overall satisfaction in adulthood [15]. Advances in reproductive medicine continue to enhance the prospects for fertility preservation (FP), offering options for women undergoing cancer treatment. Established methods for FP in post-pubertal patients include embryo and oocyte cryopreservation following hormonal stimulation before initiating therapy. Unfortunately, these methods cannot restore normal ovarian function and protect against POI. Ovarian tissue cryopreservation (OTC) is the only procedure available for prepubertal and premenarcheal girls. This method does not require prior hormonal stimulation and does not delay anti-cancer therapy. Typically, one ovary is removed laparoscopically and sliced, with the fragments gradually frozen [13]. After ovarian tissue transplantation (OTT), restoration of both reproductive and hormonal function is possible [16]; however, this typically lasts between 2 and 5 years post-transplantation [17]. The use of these methods may be limited by factors such as age, pubertal status, treatment protocol, religion, and the desire for contraception.

Enhancing existing FP methods and developing new effective therapies to safeguard fertility and protect against POI is an active area of research. Increased understanding of the molecular processes related to ovarian damage caused by chemotherapy and radiotherapy has led to the development of new therapies known as fertoprotective agents [18]. This progress may indicate a new era in which treatments protect the gonads from the harmful effects of cancer therapies. This review will focus on the molecular mechanisms of ovotoxicity from radiation, chemotherapy, and pharmacotherapeutic strategies for protecting fertility in female patients exposed to gonadotoxic therapies.

## 2. Regulation of Ovarian Reserve

A female is born with a finite number of unrenewed primordial follicle (PMF) pools which determine female reproductive longevity throughout her lifetime. Each PMF comprises a single layer of squamous granulosa cells and a non-growing, meiotically arrested oocyte that contains the female germline. PMFs, in quiescence, await their turn for primordial follicle activation, which results in recruitment into the growing pool of preantral follicles. After this, a small proportion develops further into antral follicles. This process, known as initial or continuous recruitment, is a natural part of ovarian follicle development and leads to the atresia of follicles at various stages of maturation, resulting in a decrease in the total number of follicles in the ovaries. At menopause, the number of remaining PMFs is less than 1000 [19].

Cyclic recruitment of antral follicles begins with the onset of menstrual cycles following puberty. A proper neuroendocrine balance within the HPO axis and local intraovarian factors is essential during the menstrual cycle. The gonadotropin-releasing hormone (GnRH) pulse generator in the hypothalamus triggers the pituitary gland to release two key hormones: FSH and luteinizing hormone (LH). Under the influence of FSH, a select group of antral follicles is chosen for further development. Among these, one follicle grows more rapidly than the others. As this dominant follicle matures, it produces elevated levels of estrogen and inhibins, which subsequently suppress circulating FSH levels. This suppression results in the atresia of smaller, less responsive follicles. Consequently, only the dominant follicle continues its development, progressing to the preovulatory phase and ultimately releasing an oocyte during ovulation.

As ovarian reserve declines with age, lower levels of inhibins and estrogen reduce negative feedback on the pituitary, resulting in increased FSH levels over time. This elevation in FSH during the luteal–follicular transition can lead to faster selection of the dominant follicle and ovulation, potentially resulting in shorter or irregular menstrual cycles.

**Figure 1:**
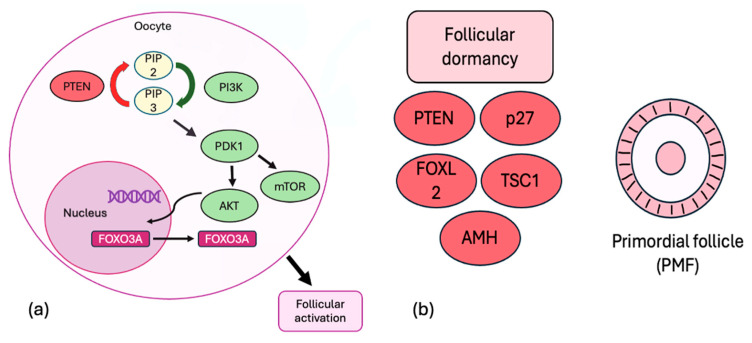
(**a**) The PI3K complex, Akt, and mTOR pathways are essential for follicular activation. The PI3K complex converts PIP2 to PIP3, which activates PDK1 and Akt. Akt then moves to the nucleus to phosphorylate FOXO3A, causing it to relocate to the cytoplasm and trigger follicular activation. PTEN negatively regulates the PI3K/Akt pathway by converting PIP3 to PIP2, acting as a natural brake against overactivation. (**b**) PMFs’ quiescence is maintained by molecules such as PTEN, TSC1–TSC2, FOXO3A, p27, AMH, and FOXL2.

Research using genetically modified mouse models enhanced the understanding of the molecules that are essential for maintaining follicular quiescence and survival. The phosphoinositide 3-kinase (PI3K) complex, protein kinase B (Akt), and mammalian target of rapamycin (mTOR) pathways are crucial for initiating follicular activation. The PI3K complex phosphorylates phosphatidylinositol-4,5-bisphosphate (PIP2) into phosphatidylinositol-3,4,5-triphosphate (PIP3). PIP3 subsequently activates pyruvate dehydrogenase kinase 1 (PDK1) and Akt. Following activation, Akt translocates to the nucleus, where it phosphorylates forkhead box protein O3A (FOXO3A). This process causes FOXO3A’s relocation to the cytoplasm, resulting in the triggering of oocyte activation. The quiescence of PMFs is supported by various molecules, including phosphatase and tensin homolog deleted on chromosome 10 (PTEN), tuberous sclerosis complexes 1–2 (TSC1–TSC2), forkhead box protein O3A (FOXO3A), p27, anti-Müllerian hormone (AMH), and forkhead box L2 (FOXL2). PTEN acts as a negative regulator of the PI3K/Akt signaling pathway by dephosphorylating PIP3 to PIP2, serving as a natural “brake” to prevent its overactivation [20,21] (Figure 1).

Additionally, many studies suggest that autophagy plays an important role in preserving PMFs by protecting oocytes from oxidative stress and apoptosis [22,23,24]. The mTOR signaling pathway inhibits autophagy, in contrast to the FOXO3A transcription factor, which induces expression of autophagy genes [25]. However, the precise mechanisms of inducing autophagy are not clearly understood.

Measuring PMFs, which comprise over 99% of the total follicle pool, is an objective method for assessing ovarian reserve. However, histological examination remains the only way to detect PMFs. Therefore, the antral follicle count (AFC) and AMH are the most commonly used and accessible clinical tools, due to their low intercycle variation and ease of measurement [13]. However, these methods can be affected by several factors, such as recent chemotherapy, ovarian surgery, pregnancy, hormonal contraception use, or smoking [26].

## 3. Molecular Mechanism of Gonadotoxicity of Radiation and Chemotherapy

Apoptosis and atresia of growing follicles play critical roles in the ovotoxic effects of chemotherapy and radiotherapy; PMFs are highly sensitive to radiation, with a lethal dose estimated to be less than 2 Gy for 50% of the population [27].

Chemotherapeutic agents can be categorized into alkylating agents, platinum-based drugs, taxanes, antitumor antibiotics, and antimetabolites. Alkylating agents such as Cy, busulfan, and dacarbazine; platinum complexes including Cs and carboplatin; and taxanes like paclitaxel are highly gonadotoxic. Extensive research on animal models and histological analyses of human ovarian segments following chemotherapy exposure have identified several mechanisms of ovarian damage. Cy and its active metabolites induce DNA double-strand breaks (DSBs) that block DNA replication and trigger apoptosis [28]. Both alkylating agents and platinum drugs induce cell apoptosis by creating DNA cross-links, leading to DNA breaks [29]. Taxanes stabilize microtubules and inhibit their disassembly during mitosis, thereby arresting cell division. The anthracycline antibiotic doxorubicin (DXR) inhibits DNA function, leading to the accumulation of DNA double-strand breaks (DSBs), which triggers apoptosis in stroma and granulosa cells. Doxorubicin also stimulates the production of oxygen-free radicals, resulting in mitochondrial dysfunction and further cellular damage [30].

**Figure 2:**
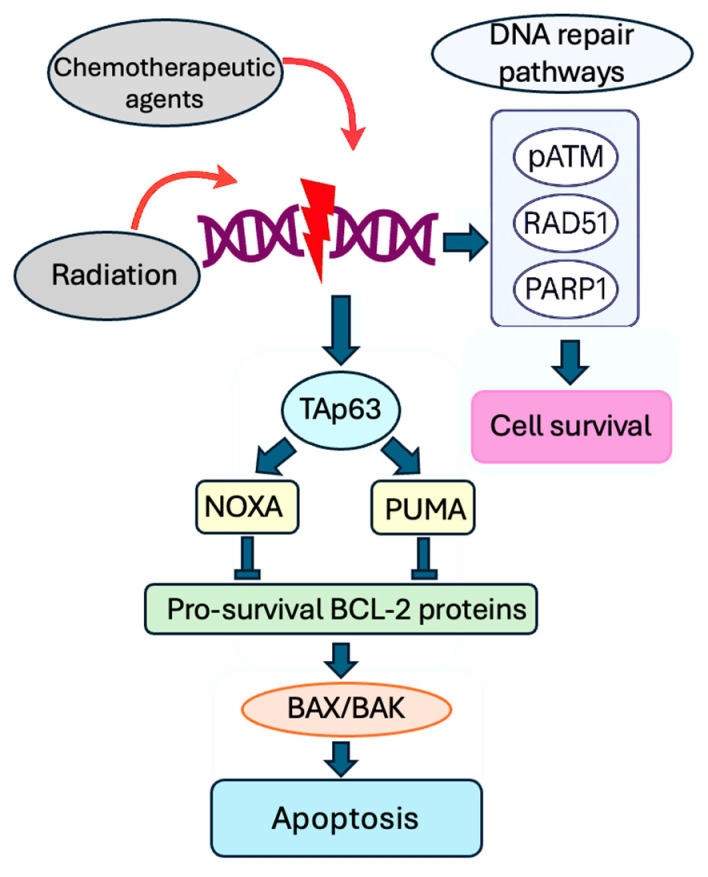
Molecular mechanism of gonadotoxicity from radiation and chemotherapy. Chemotherapeutic agents and radiation cause DNA alterations, mainly in DSBs, leading to cell death through apoptosis or the activation of repair pathways for cell survival: pATM, RAD51, or PARP1. If the repair pathways are insufficient, DNA damage triggers apoptosis via TAp63, PUMA, and NOXA, activating proteins such as BAX/BAK.

Molecules used in chemotherapy and radiation induce alterations in DNA, primarily in DSBs, which may lead to either cell death through apoptosis or the activation of DNA repair pathways, allowing for cell survival [31]. Radiation induces DNA damage via direct linear energy transfer and indirectly by generating reactive oxygen species [32]. The DNA repair pathways vary based on several factors, including the specific chemotherapeutic agents used, and may involve proteins such as pATM, RAD51, or PARP1. DNA damage triggers cellular apoptosis when these repair pathways are not adequately activated. This mechanism is initiated by TAp63, the p53-upregulated modulator of apoptosis (PUMA), and the Phorbol-12-myristate-13-acetate-induced protein 1 (NOXA), which subsequently bind to pro-survival BCL-2 family member proteins, which causes activation of BCL2-associated X protein (BAX) and BCL2-antagonist/killer (BAK), leading to apoptosis [33] (Figure 2).

Multiple hypotheses have been proposed to elucidate the gonadotoxic mechanisms, including direct toxicity to PMFs, leading to DNA damage and apoptosis, as well as indirect depletion of PMFs through over-recruitment (the “burn-out” theory). However, it remains unclear whether apoptosis directly affects the loss of PMFs that comprise the ovarian reserve.

**Figure 3:**
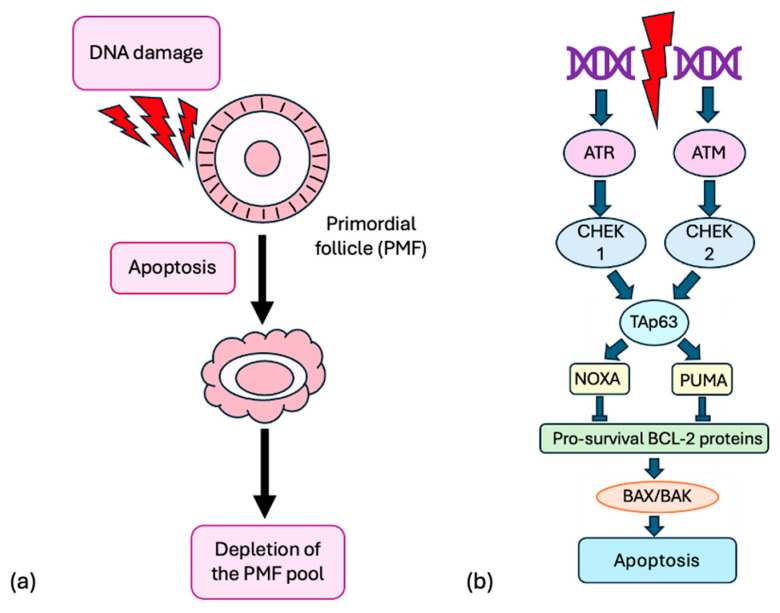
(**a**) DNA damage leads to the direct apoptosis of PMFs, resulting in the depletion of the follicular reserve. (**b**) DNA damage activates ATM and ATR pathways, resulting in the phosphorylation of CHK2 and CHK1, which leads to apoptosis via Tap63, PUMA, and NOXA, activating proteins such as BAX/BAK.

Many studies strongly support the idea that direct damage to PMFs leads to acute and significant depletion of the PMF reserve due to DNA damage, which activates the ataxia telangiectasia mutated (ATM) kinase and ataxia-telangiectasia-and-Rad3-related (ATR) pathways. This results in the phosphorylation of checkpoint kinases 2 (CHK2) and 1 (CHK1), leading to the activation of Tap63 (Figure 3) [34,35,36].

Nguyen et al. confirmed the role of PUMA, a pro-apoptotic protein, along with its transcriptional activator, TAp63, in the apoptotic elimination of PMFs in mice exposed to chemotherapy. The absence of PUMA protected PMFs from apoptosis induced by both Cy and Cs. However, knocking down TAp63 protected the ovarian reserve only after Cs treatment, suggesting that different chemotherapy agents may activate PUMA to varying extents [37]. Moreover, PMFs have higher expression of TAp63 than growing ones; thus, PMFs are more likely to undergo DNA-damage-induced apoptosis [38].

**Figure 4:**
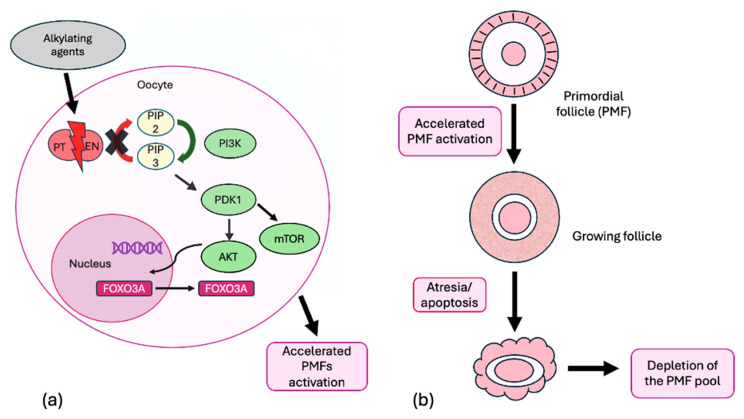
(**a**) The alkylating agent disrupts PTEN, leading to the overactivation of the PI3K signaling pathway and an increase in the phosphorylation of key proteins, Akt, mTOR, and FOXO3A, resulting in accelerated PMFs activation. (**b**) The accelerated recruitment of PMFs into the pool of actively growing follicles, which subsequently undergo atresia or apoptosis, leads to the depletion of the follicular reserve.

The “burn-out” theory suggests that anti-cancer drugs stimulate the activation of follicles from the quiescent pool, resulting in accelerated atresia and depletion of the PMF reserve [39,40,41]. In vivo animal models have shown that agents like Cy can induce the “burn-out” of PMFs by disrupting PTEN, thus overactivating the PI3K signaling pathway and leading to increased phosphorylation of key proteins Akt, mTOR, and FOXO3A (Figure 4).

This process inhibits autophagy and enhances the recruitment of PMFs into the pool of actively growing follicles, which later undergo atresia or apoptosis, reducing AMH secretion and further amplifying follicular activation and the subsequent depletion of the follicular reserve. In a study conducted by Lande et al., the activation and loss of PMFs were also observed in human ovarian tissue exposed in vitro to phosphoramide mustard, a Cy metabolite [41]. This is the only research on a human model to date that has proved Cy-induced PMF activation.

On the other hand, the burn-out hypothesis remains speculative and is a minor factor in the depletion of PMFs, according to many studies. If chemotherapy were to induce the widespread activation of PMFs, a subsequent wave of follicle depletion would need to occur, either through the direct cytotoxicity of growing follicles or physiological atresia. None of the studies supporting the burn-out theory have quantitatively demonstrated significant losses in the primary or secondary follicle populations to corroborate this proposed sequence. Moreover, if a substantial subset of these prematurely activated follicles advanced through the growth trajectory, an increase in ovulatory follicles should be detectable in animal models weeks after treatment [42]. The study by Meirow et al. showed no increase in the number of corpora lutea up to 4 weeks [43]. Goldman et al. demonstrated an increase in primary follicle density in the mice model 24 h following administration of a single dose of Cy [44], even though at least 7 days were required for transitions from primordial to primary stages in mice in vivo [45].

All studies supporting the burn-out theory were conducted on rodent models, except for one involving a human model. In contrast, Devos et al. did not observe significant activation of the PI3K/PTEN/Akt pathway in human ovarian tissue after chemotherapy [46]. Furthermore, these pathways were suppressed in PMFs 12 h after Cy administration in a human xenograft model [47]. On the other hand, the PI3K/PTEN/Akt pathway is universal and is not limited to germ cells; its activation due to chemotherapy may be related to the cell survival response [48]. The reasons why only specific PMFs are activated while others remain quiescent are still uncertain, as is why activated follicles would die instead of starting to grow, resulting in hyperstimulation of the ovaries following cytotoxic intervention. The limitations of studies suggesting the’ burn-out’ mechanism include merging primordial and transitional follicles and categorizing them as ‘non-growing’ or using whole ovary extracts to evaluate the activation of the PI3K/Akt/mTOR pathway. Furthermore, histological findings differ across these studies. Examining entire mouse ovaries would yield a more precise assessment of follicle dynamics and clarify the distinction between activation and selective depletion [49].

As stated above, the precise mechanisms by which chemotherapeutic agents lead to the loss of the PMF reserve are not fully understood and remain an area of active investigation. The drug type, concentration, and target cell type are specific to the timing of apoptosis initiation. Different mechanisms of death could be triggered by varying drug concentrations [50]. Only establishing the dose for each chemotherapy agent that induces the equivalent effect on the given cell type will allow for the comparison of study results and extrapolating them from mice to humans [42].

Additionally, stromal damage and focal infarcts resulting from reduced blood supply due to chemotherapy and radiation may indirectly contribute to the loss of PMFs and increased follicular atresia. Furthermore, both alkylating and non-alkylating drugs can affect ovarian stromal function, leading to decreased estradiol production. This implies that even chemotherapeutics that do not influence ovarian reserve may impact ovarian endocrine function [51].

Investigating the molecular mechanisms behind the ovotoxic effects of chemotherapy and radiation has revealed targets for fertoprotective agents to reduce or prevent follicular atrophy and the depletion of the PMF pool (Table 1).

## 4. Fertoprotective Agents

- Gonadotropin-releasing hormone (GnRH)

GnRH agonists (GnRHa) stimulate GnRH receptors and trigger the release of gonadotropins, leading to the desensitization of GnRH receptors and decreased FSH secretion approximately three weeks after administration. Low levels of FSH may prevent damage to early growing follicles; however, gonadotoxicity can occur even in the absence of FSH [87]. Reduced estrogen levels result in decreased ovarian perfusion, delivering fewer ovotoxic molecules to the ovaries. Vascular effects and the upregulation of anti-apoptotic molecules such as S1P might protect against ovarian damage and fibrosis [88,89,90]. Although the precise protective mechanism of GnRHa against chemotherapy is not fully understood [91], over 20 meta-analyses have investigated the gonadal protection provided by long-acting GnRHa (leuprolide, triptorelin, and goserelin) administration during chemotherapy; however, there is a lack of evidence for improvement in ovarian reserve markers, and its efficacy remains debatable. A recent population-based cohort study conducted in Sweden, involving 24,922 women diagnosed with cancer, indicated that co-treatment with GnRHa during cancer therapy does not enhance or preserve fertility following cancer treatment. Notably, only 1.5% of the women in the study received GnRHa, and fertility outcomes were assessed solely through childbirth rates [92].

Most trials were small, retrospective, and unblinded randomized studies involving female patients with breast cancer, with only a few including solid tumors or hematological malignancies. Another weakness of the existing studies is the absence of objective methods to assess the degree of gonadal damage. Most studies focus on the incidence of POI and the level of AMH because these factors can be measured easily and accurately without requiring extensive follow-up. The search for an effective indicator of POI remains challenging. While amenorrhea and elevated FSH levels signify the end stage of this condition, they highlight the need for earlier detection methods to identify ovarian follicle loss before reaching this critical point. Additionally, researchers provided insufficient data on the desire for pregnancy, post-treatment pregnancy rates, and live births following GnRHa treatment. Fertility issues can arise from various factors, not solely from oncological treatments. Thus, to determine whether GnRHa therapies decrease the likelihood of POI and increase the chance of pregnancy, broader studies with long-term follow-ups are essential.

A breakthrough in the research to date is the study that has already commenced in Sweden: a phase III prospective, randomized, double-blind, placebo-controlled clinical trial involving 500 female participants aged 14 to 42 years, including 300 subjects diagnosed with breast cancer and 200 diagnosed with lymphoma, acute leukemia, and sarcomas. The study group receives treatment with GnRHa (triptorelin) during their gonadotoxic chemotherapy. Follow-up is scheduled for 1 year and 5 years after treatment completion. The primary endpoint will be the change in AMH at the 12-month follow-up after treatment compared to AMH levels at the end of treatment between the GnRHa and placebo groups in women with breast cancer [93].

Nevertheless, while clinical evidence regarding the efficacy of fertility protection from this treatment remains debated and is still under investigation, the safety of this approach has been established. According to the ESHRE 2020 guidelines, GnRHa should be recommended for all young women requiring chemotherapy during treatment; however, it cannot be considered a primary option for FP instead of cryopreservation techniques. Additionally, using GnRHa during chemotherapy helps prevent heavy menstrual bleeding, which may be particularly beneficial for patients undergoing treatments with high bone marrow toxicity [13].

- Anti-Müllerian hormone (AMH)

AMH is a glycoprotein hormone produced exclusively by granulosa cells in preantral and small antral follicles until dominance selection occurs, which may also potentially reduce chemotherapy-induced gonadotoxicity. In both in vivo and in vitro mouse models, AMH limited the activation of PMFs [52,53,54,55]. Superphysiological doses of AMH limited PMFs loss induced by Cy, doxorubicin, or Cs in mice [55]. Sonigo et al. evaluated the protective effect of recombinant AMH in pubertal mice treated with Cy. They provided data showing that in mice treated with concomitant injections of Cy and AMH, the number of PMFs and early growing follicles was similar to the control group, contrary to the depletion of PMFs in Cy-treated mice. They also demonstrated that AMH inhibited phosphorylation of FOXO3 and therefore induced autophagy [57]. These results are consistent with a recent study on prepubertal mice, which concluded that AMH protects the PMF pool by inhibiting follicular activation and inducing autophagy, both mediated by FOXO3A signaling [58]. Roness et al. not only confirmed reduced follicle activation and PMFs loss in pubertal mice treated with Cy and recombinant AMH but also reported that AMH does not interfere with the therapeutic actions of chemotherapy [59]. Interestingly, pretreatment with modified RNA encoding AMH (ModRNA-AMH) before Cy exposure preserved PMFs and mitigated ovarian damage by restoring gene expression and follicular growth in mice [60]. As AMH is produced and acts through receptors expressed only by the ovaries, it might serve as a targeted therapy without risk of disrupting physiological mechanisms or compromising the efficacy of chemotherapy.

- Melatonin

Melatonin (N-acetyl-5-methoxytryptamine) is commonly used in various biological processes, such as treating insomnia. It mitigates the adverse effects of radiation by stimulating antioxidant enzymes, suppressing pro-oxidant enzymes, and reducing inflammatory responses [61]. It has been proposed that melatonin acts as an antioxidant through receptor-independent and receptor-dependent mechanisms, resulting in decreased levels of pro-inflammatory cytokines (TNF-α, IL-1β, and IL-6) and by inhibiting the nuclear factor kappa B (NF-κB) signaling pathway. Therefore, it inhibits apoptosis via a caspase-dependent pathway [62]. In Cs-treated mice, melatonin suppressed activation of the PI3K-Akt-FoxO3a signaling pathway and, therefore, stopped follicular activation [63,64,65]. In a study conducted on rats by Shedid et al., the administration of melatonin after whole-body irradiation reduced oxidative stress biomarker levels such as Malondialdehyde (MDA), Total Antioxidant Capacity (TAC), and Protein Carbonyl (PCO) in the ovaries. They also confirmed previous findings that melatonin reduces inflammatory cytokines (CRP and IL-6) and caspase-3 while increasing the anti-inflammatory cytokine IL-10 levels in the serum of irradiated rats [66].

- Imatinib

Imatinib, a competitive inhibitor of tyrosine kinases activated in many cancers, is used to treat patients with chronic myeloid leukemia or gastrointestinal stromal tumors. It specifically targets c-Abl kinase, which is crucial for activating TAP63 transcriptional activity in the apoptotic pathway associated with DNA damage. Imatinib may prevent Cs-induced accumulation and activation of TAP63, leading to follicle apoptosis. Gonfoni et al. observed protection of primordial and primary follicles in the ovaries of mice treated with both Cs and imatinib, in contrast to the significant reduction in these follicles when only Cs was administered [73]. Subsequent studies confirmed similar effects [74,75]. However, it is essential to ensure that imatinib does not compromise Cs’s antitumor activity by affecting the apoptotic pathway.

Nevertheless, other research yielded different outcomes: imatinib did not protect PMFs from Cs-induced cell death nor preserve fertility [76,77]. Given these contradictory findings, further studies are necessary to evaluate whether imatinib might serve as an effective treatment to mitigate Cs’s gonadotoxic effects. Furthermore, a case report indicated that prolonged imatinib use may lead to POI [96], and another case revealed a significantly reduced ovarian response to exogenous gonadotropin stimulation, which normalized after discontinuation of imatinib [97]. More studies are needed to assess the safety and efficacy of imatinib based on different exposure times and doses.

- Granulocyte colony-stimulating factor (G-CSF)

G-CSF is a glycoprotein that stimulates the bone marrow to produce granulocytes and stem cells. However, the administration of G-CSF into ischemic tissue has been confirmed to improve neovascularization [98]. Due to vascular damage induced by chemotherapy and radiation, G-CSF has been tested as a fertoprotective agent. Treatment with G-CSF and vascular endothelial growth factor (VEGF) has decreased chemotherapy-induced ovarian follicle loss and extended the time to POI in female mice treated with Cy and busulfan [94]. In another study, follicle counts and serum AMH levels were significantly increased in mice treated with Cs and G-CSF compared to those treated with Cs alone, confirming this fertoprotective effect [95].

However, the use of VEGF may present challenges because of its role in cancer angiogenesis, while G-CSF has been shown to promote neovascularization after ischemia in various organs, including the brain and heart [99].

- Sphingosine-1-phosphate (S1P) and ceramide-1-phosphate (C1P)

Sphingolipids S1P and C1P are membrane sphingolipids that regulate cell fate and play opposing roles in apoptosis and survival [100]. S1P inhibits pro-apoptotic signaling pathways initiated by ceramide and promotes the survival of PMF by modulating caspase activation and preserving mitochondrial integrity. S1P has been extensively studied for its capacity to prevent oocyte apoptosis induced by radio/chemotherapy. Kaya et al. demonstrated that administering S1P into the ovarian bursa before whole-body irradiation resulted in a lower percentage of apoptotic cells, primarily in PMFs; however, it was ineffective in preventing apoptosis in rats treated with intraperitoneal Cy [78]. In rat models, Zhao et al. confirmed that S1P protects against radiation-induced ovarian injury. Administering S1P helped maintain higher AMH levels and preserved ovarian structure and follicle count compared to radiation exposure in the lower abdominal reproductive system without S1P. The research also identified specific mitochondrial-related genes (UQCRH, MICU2, and GPX4) that were differentially expressed following radiation exposure and S1P treatment [79]. Another study on cultured ovarian granulosa cells treated with S1P showed a protective effect against radiation-induced ferroptosis. S1P upregulated glutathione peroxidase 4 (GPX4), an essential enzyme that reduces lipid peroxides and prevents ferroptosis. By enhancing the expression of GPX4, S1P helped maintain cellular homeostasis and protected cells from oxidative damage [80]. However, the study specifically focused on ovarian granulosa cells, which limits the generalizability of the findings to other cell types. Li et al. conducted the first study on human ovarian xenografts, confirming that sphingosine-1-phosphate (S1P) can prevent apoptosis in human ovarian follicles induced by Cy and doxorubicin [81]. This research provided the first opportunity to evaluate the protective effects of S1P in a setting that closely resembles human physiology. The findings lay the groundwork for further studies to investigate the long-term effects of S1P on ovarian function and its safety in humans. Although S1P was administered locally, it exhibited systemic effects, as evidenced by its impact on the ovaries of xenografted mice. This raises concerns about the potential systemic side effects of S1P. While S1P did not diminish the effectiveness of chemotherapy on specific cancer cells in rodent models, similar data in human models are still lacking. It is essential to ensure that S1P does not interfere with the efficacy of cancer treatments. Additionally, S1P has been shown to reduce the atresia of PMFs during the slow freezing and thawing of human ovarian cortical strips [82].

The administration of C1P helped maintain the number of primordial, primary, and preantral follicles in the ovaries of mice treated with Cy. It decreased the expression of pro-apoptotic markers such as BAX and increased anti-apoptotic markers like BCL-XL in the ovaries [83].

One significant limitation of these treatments is that they must be administered directly into the ovaries or continuously. However, a long-acting oral form of an S1P analog, the P S1P analog (FTY720, fingolimod), has recently been developed for women with multiple sclerosis. A study on groups of rats confirmed that it may decrease spontaneous follicular apoptosis, as indicated by the higher ratios of non-apoptotic PMFs and the levels of AMH in the treated rat groups compared to the placebo group [84].

Although substantial evidence shows that C1P and S1P protect the ovarian reserve from radiation and chemotherapy damage, long-term studies are needed that include broader gene analysis and an exploration of the mechanisms involved in the protective effects of C1P and S1P. Most importantly, validation in other animal models and, eventually, clinical trials is required.

- mTOR Inhibitors

Everolimus (RAD001), a clinically approved drug, and the experimental drug INK128 inhibit the mTOR pathway and may preserve the ovarian reserve [44]. Everolimus reduced the gonadotoxicity induced by Cs in an in vivo mouse model [67]. Rapamycin is another mTOR inhibitor and antitumor agent. Tanaka et al. demonstrated that rapamycin reduced Cy-ovarian follicle loss in a breast cancer xenograft mouse model and did not interfere with antitumor effects [68]. Rapamycin could also be used alongside hormone therapy, recombinant anti-Müllerian hormone [69], or a GnRHa, offering better follicle protection against high doses of Cy. This treatment may provide a compelling option for FP during conventional chemotherapy.

Moreover, incorporating rapamycin during OVT and subsequent autotransplantation (OTCTP) enhanced fertility restoration in a murine orthotopic transplantation model while reducing the excessive activation of PMFs during grafting. This improvement may increase the chances of successful pregnancies after OTCTP procedures [70].

- AS101

AS101 [ammonium trichloro (dioxoethylene-o,o’) tellurate] is an immunomodulatory compound that modulates the PI3K-Pten-Akt pathway [101]. This molecule was tested to prevent Cy-induced follicle loss, as it was found to activate the PI3K pathway that may induce PMF recruitment and subsequent depletion of the ovarian reserve [71]. In vivo treatment of mice with AS101 reduced Cy-induced follicular depletion. Moreover, no increase in fetal malformation was observed in mice previously treated with AS101, indicating the safety for offspring. An additional investigation involving the natural carotenoid crocetin alongside AS101 in mice exposed to Cy corroborated the downregulation of the PI3K/AKT/FOXO3A signaling pathway, which plays a role in follicle activation [72].

- Luteinizing hormone (LH)

In a study conducted by Rossi et al., LH inhibited PMF pool depletion in prepubertal mice treated with Cs. LH activated anti-apoptotic signals, reduced the oocyte levels of the pro-apoptotic TAp63 protein, and promoted the DNA repair pathway in the oocytes [85]. In a study by Lamsira et al., ovarian cortical tissues were exposed to phosphoramide mustard (PM), the active metabolite of Cy. It was shown that in the group treated with LH, there was a reduction in the levels of pro-apoptotic factors such as PUMA and cleaved caspase 3, as well as modulation of follicle activation by lowering the AKT-FOXO3a signaling axis [86]. These findings support the consideration of LH as a potential fertoprotective agent in clinical trials.

## 5. Conclusions

Fertoprotective agents could be combined with currently available FP methods without restrictions based on age, treatment, or religion. Additionally, they would protect not only against the loss of reproductive ovarian function but also against the loss of hormonal function, significantly enhancing the quality of life for young patients after oncological treatment.

Although our understanding of the mechanisms behind gonadotoxic therapies has improved, significant research gaps remain regarding their reliance on mouse models, the lack of suitable experimental models replicating human ovarian dynamics, and the complex molecular and endocrine pathways regulating follicular reactions to cancer treatments. While mouse models provide valuable insights in reproductive research, the differences in ovarian structure and physiology between humans and mice are considerable. Human ovaries are larger and denser, featuring a monoovulatory menstrual cycle, whereas mouse ovaries are smaller, less dense, and follow a polyovulatory, short estrous cycle. Therefore, caution is paramount when interpreting data obtained from mouse models. It is crucial to approach data derived from these models with care. Long-term studies encompassing broader analyses and exploring the mechanisms involved in the protective effects are necessary. Most importantly, validation in other animal models and, eventually, clinical trials is essential.

Additionally, long-term observation is essential to confirm that these substances do not reduce the anti-cancer efficacy of oncological treatments. It must also be ensured that they do not cause permanent DNA alterations in granulosa cells, which could increase the risk of miscarriages, infertility, or genetic and developmental abnormalities in the fetus.

## Figures and Tables

**Table 1:** Protective mechanisms of fertoprotective agents assessed in either rodent models or both human and rodent models.

Fertoprotective Mechanism	Fertoprotective Agents	Mechanism of Action	Model	References
Inhibition of primordial follicle recruitment	Anti-Müllerian hormone (AMH)	Ovary hormone	Rodent	[52,53,54,55,56,57,58,59,60]
	Melatonin	Pineal hormone	Rodent	[61,62,63,64,65,66]
	Everolimus	mTOR inhibitor	Rodent	[44,67]
	Rapamycin	mTOR inhibitor	Rodent	[68,69,70]
	AS101	PI3K-Pten-Akt pathway modulator	Rodent	[71,72]
Inhibition of primordial follicular apoptosis	Imatinib	Competitive tyrosine kinase inhibitor	Rodent	[73,74,75,76,77]
	Sphingosine-1-phosphate (S1P)	Membrane sphingolipid	Rodent	[78,79,80,81]
			Human ovarian cortical strips (in vitro)	[82]
	Ceramide-1-phosphate (C1P)	Membrane sphingolipid	Rodent	[83,84]
	Luteinizing hormone (LH)	Gonadotropin	Rodent	[85]
			Human ovarian cortical strips (in vitro)	[86]
Vascular effect Upregulation of anti-apoptotic molecule	Gonadotropin-releasing hormone agonists (GnRHa)	Inhibition of the pituitary-gonadal-axis	Human (clinical trials)	[87,88,89,90,91,92,93]
			Rodent	
Vascular effect	Granulocyte colony-stimulating factor (G-CSF)	Granulocyte colony-stimulating factor	Rodent	[94,95]

## Data Availability

The data underlying this article are available in the article and, when necessary, via 73465@student.ump.edu.pl.
